# 

**Published:** 1839-04-01

**Authors:** 


### CONTENTS

OF THE

### MEDICO-CHIRURGICAL REVIEW,

### No. LX. APRIL 1, 1839,

[BEING No. XX. OF A DECENNIAL SERIES.]

### REVIEWS.

I.

1. Urinary Diseases and their Treatment. By ROBERT WILLIS, M.D.
2. On Granular Degeneration of the Kidneys, and its connexion with Dropsy, Inflammation and other Diseases. By ROBERT CHRISTISON, M.D.
3. Traité des Maladies des Reins, et des Altèrations de la Sécrétion Urinaire, étudiées en elles-memes et dans leurs Rapports avec les Maladies des Ureteres, de la Vessie, &c. Par P. RAYER, M.D. .. .. 349

1. Morbid States in which the Urine contains, in solution or as precipitates, certain Principles which do not occur in the healthy Secretion .. .. 351
   - A. Discharge of Urine which contains the Lithic Oxyde—Lithoxiduria.. .. 353
   - B. Of the Discharge of Urine which contains Cystine—Cystinuria.. .. 353
   - C. Of the Discharge of Urine which contains the Purpuric Acid and its Salts —Porphururia.. .. 353
   - D. Of the Discharge of Urine containing a Salt of the Oxalic Acid—Oxaluria, 354
   - E. Of the Discharge of Urine containing the Elements of Urea in the shape of Carbonate of Ammonia .. .. 355
   - F. Of the Discharge of Urine containing the Hydrocyanic & Ferrocyanic Acid 357
   - G. Of the Discharge of Urine containing Carbonate of Lime in solution or as a deposite .. .. 358
   - H. Of the Discharge of Urine which is luminous or phosphorescent .. .. 358
   - I. Of the Discharge of Urine of various abnormal and remarkable Colours .. 359
   - K. Of the Discharge of Urine of various peculiar and abnormal Odours.. .. 360
   - L. Of the Discharge of Urine containing Silica in solution or as a deposite .. 361
2. Morbid States in which certain Matters, being Constituents of the Blood, are contained in the Urine.. .. 361
   - A. Of the Discharge of Urine, having the Albumen of the Blood mingled with it—Sero-albuminous Urine.. .. 362
   - B. Symptoms and History .. .. 372
   - C. Secondary Diseases.. .. 381
   - D. Causes .. .. 388
   - E. Prognosis.. .. 390
   - F. Treatment .. .. 392

II.

Recherches Anatomiques sur l'Emphyseme Pulmonaire. Par le Dr. LOMBARD ..
Anatomical Researches on Pulmonary Emphysema.. .. 397

1. Anatomical Details regarding Pulmonary Emphysema .. .. 397
2. Theory of the Formation of Pulmonary Emphysema .. .. 399
3. Explanation of the Symptoms of Pulmonary Emphysema by the Nature of the Anatomical Lesion .. .. 401
4. Practical Consequences respecting the Treatment of Pulmonary Emphysema .. 403

III.

An Exposition of Quackery and Imposture in Medicine, &c. By Dr. TICKNOR. With Notes by W. WRIGHT, Esq. .. .. 405

IV.

Outlines of Military Surgery. By Sir GEORGE BALLINGALL, M.D. .. .. 408

V.

I. The Cyclopædia of Anatomy and Physiology. Part XV. .. ..
II. A Treatise on the Structure, Economy, and Diseases of the Ear. By GEORGE PILCHER, Esq. .. .. 441

1. The Functions of the Ear .. .. .. .. .. .. .. .. .. .. .. .. 414

2. The Diseases of the Ear .. .. .. .. .. .. .. .. .. .. .. .. 428

VI.

A Treatise on the Medical Jurisprudence of Insanity. By J. Ray, M.D. .. .. .. .. 431
1. Mental Diseases in general .. .. .. .. .. .. .. .. .. .. .. .. 439
2. Legal Consequences of Intellectual Mania .. .. .. .. .. .. .. .. 441

VII.

The Discovery of the Vital Principle, or the Physiology of Man .. .. .. .. .. .. 443

VIII.

An Essay on the most important Diseases of Women. By Robert Fergusson, MD.. 461

1. Various Forms of Puerperal Fever .. .. .. .. .. .. .. .. .. .. 461

A. Peritoneal Form .. .. .. .. .. .. .. .. .. .. .. .. .. 462

B. Gastro-Enteric Irritation .. .. .. .. .. .. .. .. .. .. .. 463

C. Third or Nervous Form .. .. .. .. .. .. .. .. .. .. .. 464

D. Fourth or Complicated Form .. .. .. .. .. .. .. .. .. .. 464

2. Morbid Anatomy .. .. .. .. .. .. .. .. .. .. .. .. .. .. 465

3. Nature of Puerperal Fever .. .. .. .. .. .. .. .. .. .. .. 466

4. Puerperal Constitution .. .. .. .. .. .. .. .. .. .. .. .. 470

5. Treatment .. .. .. .. .. .. .. .. .. .. .. .. .. .. 472

A. Peritoneal Form .. .. .. .. .. .. .. .. .. .. .. .. 472

B. Gastro-Enteric Form .. .. .. .. .. .. .. .. .. .. .. 474

C. Treatment of the Ataxic Form .. .. .. .. .. .. .. .. .. 475

D. Treatment of the Complicated Form .. .. .. .. .. .. .. .. 475

IX.

Practical Obervations on the Causes and Treatment of Curvatures of the Spine. By Samuel Hare, Esq. .. .. .. .. .. .. .. .. .. .. .. .. .. 478

X.

I. Elements of the Pathology of the Human Mind. By Thomas Mayo, M.D. ..
II. Traité des Maladies Mentales. Par E. Esquirol, M.D. .. .. .. .. .. .. } 482
1. On Hallucinations .. .. .. .. .. .. .. .. .. .. .. .. .. 496
2. On the Illusions of the Insane .. .. .. .. .. .. .. .. .. .. 499

### PERISCOPE.

### Bibliographical Notices.

1. Thoughts and Observations upon Pauperism, Poor-laws, Emigration, Medical Relief, and the Prevention of Crime. By William Fergusson, M.D. .. .. .. .. 504
2. Stammering practically considered, with the Treatment in detail. By T. Bartlett, Esq. .. .. .. .. .. .. .. .. .. .. .. .. .. .. 507
3. An Improvement in the Pathology and Treatment of Small-pox, &c. By Robert Stephens, M.R.C.S. .. .. .. .. .. .. .. .. .. .. .. .. 509
4. The Philosophy of Disease. By James Bower Harrison, M.R.C.S. .. .. .. 511
5. Vital Statistics of Glasgow. By Robert Cowan, M.D. .. .. .. .. .. .. 511
1. Statistics of Fever and Small-pox prior to 1837 .. .. .. .. .. .. 511
2. Statistics of Fever for 1837 .. .. .. .. .. .. .. .. .. .. 515
3. Remarks suggested by the Mortality Bills .. .. .. .. .. .. .. 516
6. Elements of Physiology. By J. Muller, M.D. Translated by William Baly, M. D. 517
7. Illustrations of Cutaneous Disease. A Series of Delineations of the Affections of the Skin in their more interesting and frequent forms: with a practical Summary of the Symptoms, Diagnosis, and Treatment. By Robert Willis, M.D. .. .. 519
8. Illustrations of Osteology. By Theodore G. Boisragon, M.D. .. .. .. .. 520
9. A History of British Birds. By William Yarrell, F.L.S. Part XI. .. .. .. 521
10. A History of British Reptiles. By Thomas Bell, F.R.S. .. .. .. .. .. 521
11. The Naturalist's Library. Conducted by Sir William Jardine, Bart. Mammalia. Vol. III. .. .. .. .. .. .. .. .. .. .. .. .. .. .. 522
12. The Surgeon's Vade Mecum, a handbook of the Principles and Practice of Surgery. By Robert Druitt, M.R.C.S. .. .. .. .. .. .. .. .. .. .. 522
13. A general Outline of the Animal Kingdom. By Thomas Rymer Jones, F.Z.S. Parts III. IV. and V. .. .. .. .. .. .. .. .. .. .. .. .. 522
14. Elements of Chemistry, including the recent Discoveries and Doctrines of the Science. By the late Edward Turner, M.D. .. .. .. .. .. .. .. } 524
Part III. Organic Chemistry. By Professor Liebig.. .. .. .. .. .. ..

15. The Quarantine Laws. Mr. Holroyd's Letter to Sir J. C. Hobhouse on the Abuses and Inconsistencies of the said Laws .. .. .. 525

16. The Medical Portrait Gallery .. .. .. 525

1. The Death of Vesalius .. .. .. 526

2. Abernethiana .. .. .. 526

3. Memoir of William Hunter .. .. .. 527

4. Baillie's Generosity .. .. .. 528

5. Advantages of Royalty in Death-bed Scenes .. .. .. 528

17. A Letter to Dr. Chambers on several important Points relating to the Nature and Treatment of Gout. By Sir Charles Scudamore, M.D. .. .. .. 530

18. Anatomie comparée du Système Nerveux consideré dans ses Rapports avec l'Intelligence. Par Fr. Leuret, M.D. .. .. ..

Atlas, Première Livraison .. .. .. } 532

### Spirit of the British and American Periodicals.

1. Virtues of the Iodide of Potassium .. .. .. 533
2. State of the Faculty and of Physic in Algiers .. .. .. 534
3. Dr. Corrigan's new mode of effecting the Inhalation of Iodine, &c. in Pulmonary Diseases .. .. .. 535
4. Urea in the Blood in Cholera .. .. .. 536
5. Mr. Smee on Moulding Tablets for Fractures .. .. .. 536
6. Observations on the Efficacy of the Salts of Silver in the Treatment of Secondary Syphilis. By William Johnston, M.D., and James Trudeau, M.D. .. .. .. 538
7. Observations on various Diseases. By Robert Graves, M.D. .. .. .. 539
1. Neuralgia of the Larynx .. .. .. 539
2. Neuralgia of the Testicle .. .. .. 539
3. Gout and Sinapisms.. .. .. .. 540
4. Mr. Cusack's mode of applying the Nitrate of Silver to Enlarged Amygdalæ 541
8. Dr. Law on the Exhibition of Mercury in Minute Doses .. .. .. 541
9. Shortening of the Neck of the Thigh Bone independently of Fracture, in Early or in Middle Life. By George Gulliver, Esq. .. .. .. 543
10. Cases of Anomalous Aneurysm of the Aorta, resulting from Effusion of Blood between the Laminæ composing the Middle Coat of that Vessel. By C. W. Pennock, M.D. .. .. .. 545
11. Account of a Case of Dissecting Aneurysm, seen at an early stage. By Paul B. Goddard, M.D. .. .. .. 545

### Clinical Review, and Hospital Reports.

ST. BARTHOLOMEW'S HOSPITAL.
1. Observations of Mr. Lawrence on Gonorrhœal Rheumatism and Gonorrhœal Ophthalmia.. .. .. .. 549
2. Clinical Observations in Surgery. By M. R. Smith, M.D. .. .. .. 550
1. Extirpation of the Parotid Gland .. .. .. 550
2. Successful Amputation for Traumatic Gangrene .. .. .. 552
MASSACHUSSETT'S GENERAL HOSPITAL.
3. Cases of Pneumo-thorax, and Experiments to determine the Causes of Metallic Tinkling. By J. Bigelow, M D... .. .. .. 552
4. Clinical Reports from the Pennsylvania Hospital .. .. .. 554
1. Dislocation of the Humerus, reduced at the end of 21 days .. .. .. 554
2. Dislocation of the Head of the Radius, backwards, reduced .. .. .. 555
3. Three Cases of Compound Fracture of the Cranium .. .. .. 555
4. Mortality after Amputations in the Pennsylvania Hospital .. .. .. 556
5. Statistical Report of the Richmond Lunatic Asylum. By John Mollan, M.D. .. 558
6. Twentieth Report of the Directors of the West Riding of York Pauper Lunatic Asylum.. .. .. .. 565
MARYLEBONE INFIRMARY.
7. On Long-continued Contraction of the Lower Extremities, from an Affection of the Spine. By R. A. Stafford, Esq... .. .. .. 567
8. Royal Infirmary of Edinburgh Clinical Report. By J. Syme, Esq. .. .. .. 572
1. Dislocation of the Thigh Bone on the Dorsum Ilii of 9 weeks' standing, reduced 572
2. Dislocation of the Thigh Bone on the Dorsum Ilii, of 6 weeks' standing, reduced 573

3. Fortunate Treatment of Fracture at a Late Period .. .. .. .. .. .. .. 573
4. Why Torn Arteries do not Bleed.. .. .. .. .. .. .. .. .. .. .. .. .. 573
5. Fistula in Ano .. .. .. .. .. .. .. .. .. .. .. .. .. .. .. 574
6. Extraordinary Operations for Hæmorrhoids in Edinburgh .. .. .. .. .. 574
9. Dundee Lunatic Asylum. Eighteenth Report of the Directors .. .. .. .. .. 575
10. Experience of Mr. Porter in Puncturing the Dura Mater after Trephining .. .. 578
11. Clinical Observations on the State of the Heart and on the Use of Wine in Typhous Fever. By William Stokes, M.D. .. .. .. .. .. .. .. .. .. .. .. .. 579

### Spirit of the Foreign Periodicals, &c.

1. On Vaccination and Small-pox .. .. .. .. .. .. .. .. .. .. .. .. .. 581
2. Notice of the German Journal Zeitschrift fur die Gesammte Medicin ; its contents 589
3. Extract from M. Gendrin's Lectures on Diseases of the Heart.. .. .. .. .. 589
4. Opening between the Ventricles of the Heart in an adult, without any Symptoms of Morbus Cœruleus .. .. .. .. .. .. .. .. .. .. .. .. .. .. .. 591
5. Cyanosis ; Origin of the Aorta from the Right Ventricle .. .. .. .. .. 591
6. Irregularities of the Subclavian Artery and Vein .. .. .. .. .. .. .. 592
7. Professor Dieffenbach on the Orthopædic Establishments in Paris .. .. .. .. 592
8. Concours for the Chair of Organic Chemistry at Paris .. .. .. .. .. .. .. 594
9. Treatment of Diptherite, or Croupy Inflammation of the Mouth .. .. .. .. 595
10. Professor Osiander on Puerperal Fever .. .. .. .. .. .. .. .. .. .. .. 595
11. Clinique of M. Bricheteau at the Hôpital Necker .. .. .. .. .. .. .. .. 598
12. Use of Caustic Issues in Phthisis Pulmonalis .. .. .. .. .. .. .. .. .. 599
13. M. Bricheteau on Typhus Fever .. .. .. .. .. .. .. .. .. .. .. .. .. 601
14. Cantharides proved to be a Contra-Stimulant or Antiphlogistic Remedy ; its depressing action on the Heart, &c.: its great utility in Inflammatory Diseases .. 603
15. Professors Oppenheim and Fricke on the Use of Iodine Injections in Hydrocele .. 605
16. On the Treatment of Hydrocele with Iodine Injections in the East Indies .. .. 608
17. Treatment of Prolapsus Uteri by Partial Suture of the Vulva .. .. .. .. .. 610
18. Dupuytren's Pommade against the falling off of the Hair .. .. .. .. .. .. 610
19. Dubois' Rules for the Treatment of Uterine Hæmorrhage .. .. .. .. .. .. 611
20. Poisoning by Arsenic successfully treated with the Tritoxide of Iron .. .. .. 612
21. Remarks on Paralysis of the Eyelids.. .. .. .. .. .. .. .. .. .. .. .. 613
22. Cases of Glanders in the Human Subject—Discussion at the Royal Academy on.. 613
23. A Consultation of MM. Rayer, Orfila and Caffe on a Case of Milky Urine. Chemical examination of the Urine and Blood, practical Remarks .. .. .. .. .. 617
24. Utility of Iron (especially the Ioduret) in some Syphilitic Affections .. .. .. 620

### Miscellanies.

1. A New Weekly Contemporary .. .. .. .. .. .. .. .. .. .. .. .. .. .. 621
2. Sir Philip Crampton's History of Medicine .. .. .. .. .. .. .. .. .. .. 626
3. Pellucid Solution of Magnesia .. .. .. .. .. .. .. .. .. .. .. .. .. .. 629
4. German Physicians .. .. .. .. .. .. .. .. .. .. .. .. .. .. .. .. 630
5. Animal Magnetism .. .. .. .. .. .. .. .. .. .. .. .. .. .. .. .. 630
6. Horse-Hair Gloves .. .. .. .. .. .. .. .. .. .. .. .. .. .. .. .. 631
7. Case of Extensive Aneurysm of the Thoracic Aorta. By Sir David T.H. Dickson, M.D. 631
Bibliographical Record .. .. .. .. .. .. .. .. .. .. .. .. .. .. .. .. 633

### Extra-Limites.

1. Biographical Memoir of Dr. James Johnson, Senior Editor of the Medico-Chirurgical Review .. .. .. .. .. .. .. .. .. .. .. .. .. .. .. .. .. .. .. 637
2. Delirium Tremens.. .. .. .. .. .. .. .. .. .. .. .. .. .. .. .. .. 643
3. Some Cases by Dr. Beemner .. .. .. .. .. .. .. .. .. .. .. .. .. .. 645
1. Case of Intro-susceptio cured by forcing Air into the Intestines .. .. .. 645
2. Post-mortem Examination of a Case of Dropsy .. .. .. .. .. .. .. .. 646
4. Remarkable State of Disease in a Physician .. .. .. .. .. .. .. .. .. 647
5. Report of the Medical and Surgical Society of Newcastle-upon-Tyne, on the Present State of the Medical Profession .. .. .. .. .. .. .. .. .. .. .. .. .. 648
6. Memorial of the Medical Officers serving on the Bengal Establishment to the Court of Directors of the East India Company .. .. .. .. .. .. .. .. .. .. 651
7. Drs. Forbes and Conolly .. .. .. .. .. .. .. .. .. .. .. .. .. .. .. 657

